# Relationship between early nutrition and deep gray matter and lateral ventricular volumes of preterm infants at term-equivalent age

**DOI:** 10.1007/s12519-022-00657-8

**Published:** 2023-01-04

**Authors:** Felicia Toppe, Tobias Rasche, Christel Weiss, Alexandra Schock, Ursula Felderhoff-Müser, Hanna Müller

**Affiliations:** 1 Department of Pediatrics I, Neonatology, Pediatric Intensive Care, and Pediatric Neurology, University Hospital Essen, University of Duisburg-Essen, Hufelandstr. 55, 45147 Essen, Germany; 2 Department of Medical Statistics, Biomathematics, and Information Processing, Medical Faculty Mannheim of Heidelberg University, Theodor-Kutzer-Ufer 1-3, 68167 Mannheim, Germany; 3 Pediatric Surgery, University Hospital Marburg, University of Marburg, Baldingerstraße, 35043 Marburg, Germany; 4 Department of Pediatrics, Neonatology and Pediatric Intensive Care, University Hospital Marburg, University of Marburg, Baldingerstraße, 35043 Marburg, Germany

**Keywords:** Deep gray matter, Lateral ventricles, Magnetic resonance imaging, Nutrition, Prematurity

## Abstract

**Background:**

The survival of preterm infants has improved over the last decade, but impaired brain development leading to poor neurological outcomes is still a major comorbidity associated with prematurity. The aim of this study was to evaluate the effect of nutrition on neurodevelopment in preterm infants and identify markers for improved outcomes.

**Methods:**

Totally 67 premature infants with a gestational age of 24–34 weeks and a birth weight of 450–2085 g were included. Clinical parameters and documented diet were collected from medical records. The nutritional analysis comprised the protein, fat, carbohydrate, and energy intake during different time spans. Brain development was assessed by determining deep gray matter (DGM; basal ganglia and thalamus) and lateral ventricular (LV) volumes as measured on cerebral magnetic resonance imaging scans obtained at term-equivalent age (TEA), and potential associations between nutrition and brain volumetrics were detected by regression analysis.

**Results:**

We observed a negative correlation between mean daily protein intake in the third postnatal week and MRI-measured DGM volume at TEA (*P* = 0.007). In contrast, head circumference at a corrected age of 35 weeks gestation (*P* < 0.001) and mean daily fat intake in the fourth postnatal week (*P* = 0.004) were positively correlated with DGM volume. Moreover, mean daily carbohydrate intake in the first postnatal week (*P* = 0.010) and intraventricular hemorrhage (*P* = 0.003) were revealed as independent predictors of LV volume.

**Conclusion:**

The study emphasizes the importance of nutrition for brain development following preterm birth.

## Introduction

The survival of preterm infants has significantly improved over the past 10 years. However, despite major advances in neonatal care, premature birth still poses a high risk of neurodevelopmental impairment, especially to very immature neonates with a gestational age of < 32 weeks. During human brain development, two time spans with particularly rapid growth, characterized by extensive cell proliferation (up to 20,000 cells per day), neuronal and glial differentiation, and synapse formation, are known [1]. The first one occurs within the early fetal stage between 10 and 18 weeks’ gestation, whereas the second one starts at 30 weeks’ gestation and ends with the second year of life [2, 3]. Thus, preterm infants are exposed to the extrauterine environment during a period critical for fetal brain maturation. Given the proven impact of neonatal intensive care management between premature birth and term on neurological outcomes, many research efforts have been undertaken to identify risk factors for neurodevelopmental impairment and modify therapeutic approaches accordingly [4].

The importance of nutrition in the improvement of postnatal brain development has been stressed for years [5]. Since insufficient nutrition of preterm infants is associated with reduced cognitive skills and impaired health, a strong focus is currently placed on the administration of an optimally composed diet [2, 5]. Hence, separate analysis of the influence of energy and macronutrient (protein, fat, carbohydrate) provision on neurodevelopment resulted in higher protein and fat intake during the first days of life.

The aim of this study was to evaluate the effect of various clinical and nutritional parameters on brain development in preterm infants and to identify potential prognostic markers for improved outcomes. We focused on the deep gray matter (DGM; basal ganglia and thalamus), a cerebral region with a high impact on motor and sensory functions, and lateral ventricular (LV) volumes, which are often increased in very immature infants. Brain volumes were measured on cerebral magnetic resonance imaging (MRI) scans obtained at term-equivalent age (TEA) and correlated with neonatal diet composition using regression analysis.

## Methods

### Patients

Totally 67 preterm infants born between March 2011 and December 2012 at the University Hospital Essen, Germany,  with a gestational age of < 32 weeks and/or a birth weight of < 1500 g were included. Gestational age was defined as time elapsed between the first day of the last menstrual period and the day of birth and confirmed by ultrasound. This retrospective study was approved by the Ethics Committee of the University Hospital Essen, Germany, and conducted in accordance with the Helsinki Declaration (1964) and its later amendments (17-7490-BO). The local ethics committee confirmed that parental consent to participate is not necessary due to the retrospective and anonymous character of this study.

A documented neonatal diet (e.g., breast milk, formula milk, fortifier) was used to retrospectively calculate the protein, fat, carbohydrate, and energy intake during distinct time periods. Preterm infants were fed according to an internal guideline based on current literature (recommendations of the European Society for Pediatric Gastroenterology, Hepatology and Nutrition (ESPGHAN)). Total daily fluid intake (enteral and parenteral) was gradually increased from 70–80 mL/kg on the first day of life to 150–170 mL/kg on the seventh day of life. The final volume was adjusted according to actual body weight and fluid balance and altered in special medical conditions, such as edema or polyuria.

Enteral feeding was administered via a gastric tube and successively increased by 20–30 mL/kg/day. Gastric residuals of up to 25% of the total meal volume were tolerated as long as no clinical signs of abdominal problems were observed. Preterm infants with a birth weight below 1500 g received 12 meals per day. After reaching a weight of at least 1800 g, the number of meals was reduced to 8 per day, and 6 meals per day were introduced with a weight of 2000 g. Prior to discharge, infants drank ad libitum. Enteral feeding was complemented by parenteral nutrition to reach the necessary energy intake of 40–60 kcal/kg/day during the first days of life and 110–135 kcal/kg/day during the further course.

Protein intake was gradually increased from 1.5–2.0 g/kg/day to 4.0–4.5 g/kg/day in infants with a weight below 1000 g or 3.5–4.0 g/kg/day in infants with a weight between 1000 and 1800 g. Further protein administration was adjusted depending on growth. Fat intake was started at 1.0 g/kg/day after birth and increased by approximately 0.5 g/kg/day until a maximum intake of 2.0–3.0 g/kg/day.

Adequate nutrition and therefore growth of preterm infants was defined by a daily weight increase of 15–20 g/kg, weekly length increase of 0.8–1.0 cm, and weekly head circumference increase of 0.5–0.8 cm (ESPGHAN recommendations).

### Methods

The following parameters were collected from medical records: gestational age at birth (weeks); weight at birth and discharge (g); and head circumference at birth, at corrected gestational age of 35 weeks, and at discharge (cm and percentile). Subsequently, the mean weekly head circumference increase until a corrected gestational age of 35 weeks and in each of the first four weeks of life (cm) was calculated. Data about the number of days with parenteral nutrition, the day of life when the feeding was completely enteral and the number of days without enteral nutrition due to abdominal problems were collected. Additionally, we classified the infants into groups with predominantly feeding of breast milk or of formula nutrition or with both breast milk and formula nutrition. Different clinical diagnoses, such as hyperbilirubinemia, cerebral and intraventricular hemorrhages, asphyxia or severe hypoxia, periventricular leukomalacia, small for gestational age (< 10th percentile), early onset infection (< 72 hours of life), late onset infection (> 72 hours of life), persistent ductus arteriosus (PDA), necrotizing enterocolitis (NEC), operation due to PDA, NEC or hernia and bronchopulmonary dysplasia, were examined.

Nutritional analysis comprised the cumulative energy (kcal/kg), protein (g/kg), fat (g/kg) and carbohydrate (g/kg) until postnatal days 14 and 28, respectively; the mean daily energy, protein, fat and carbohydrate until a corrected gestational age of 35 weeks; and the mean daily energy, protein, fat and carbohydrate in each of the first four weeks of life.

After obtaining written parental informed consent, cerebral MRI scans at TEA were performed on a 3 Tesla MR scanner (Magnetom Skyra, Siemens Healthcare, Erlangen, Germany) as previously published, and DGM as well as LV volumes were calculated on axial T2-weighted turbo spin echo sequences [6].

### Statistical analysis

The statistical analysis was conducted by our statistician (C. W.) using SAS software (release 9.4; SAS Institute Inc., Cary, NC, USA). For quantitative variables, mean values and standard deviations (SD) together with median, minimum, and maximum values are given. Qualitative factors are presented as absolute and relative frequencies. Pearson’s correlation coefficients were calculated to quantify the strength of the relationship between DGM or LV volume and different parameters. Subsequently, the simultaneous influence of nutrition and various clinical parameters (e.g., birth weight, gestational age at birth) on the dependent outcome variables DGM and LV volume was assessed by multiple regression analysis. All variables proven to be significant in the univariable analyses were considered for multiple linear regression analysis using the “selection = forward” option. The determination coefficients *R*^*2*^ are given as measures of goodness. Test results with *P* values less than 0.05 were regarded as statistically significant.

## Results

### Patients

Totally 67 preterm infants (33 females, 34 males) born in 2011 and 2012 with a gestational age of 28.9 ± 2.6 weeks [mean ± standard deviation (SD); median 29.4 weeks; range 23.6–34.4 weeks] and a birth weight of 1143 ± 364 g (mean ± SD; median 1170 g; range 450–2085 g) were included.

No infant showed congenital brain malformation, asphyxia, severe hypoxia or periventricular leukomalacia. There were 37 infants with hyperbilirubinemia needing therapy, but this was mild in each case and was treated with phototherapy. Blood exchange was not necessary. Three infants had grade I intraventricular hemorrhage, and three infants had grade II intraventricular hemorrhage. One infant showed intraventricular hemorrhage III and bleeding in the surrounding parenchyma on the right cerebral side, and another infant showed cerebellar bleeding on the right side, which was only detectable by MRI but not by sonography.

### Head circumference

Head circumference was 26.06 ± 2.78 cm (mean ± SD; median 26.50 cm; range 20.00–31.00 cm) at birth and 30.60 ± 1.79 cm (mean ± SD; median 31.00 cm; range 26.00–33.50 cm) at a corrected gestational age of 35 weeks. The weekly increase in occipitofrontal circumference during each of the first four postnatal weeks was 0.27 ± 0.33 cm (mean ± SD; median 0.0 cm; range − 0.50 to 1.00 cm) during postnatal week 1, 0.66 ± 0.38 cm (mean ± SD; median 0.50 cm; range 0.0–1.50 cm) in postnatal week 2, 0.80 ± 0.43 cm (mean ± SD; median 1.00 cm; range 0.0–1.50 cm) in postnatal week 3, and 0.87 ± 0.42 cm (mean ± SD; median 1.00 cm; range 0.0–2.00 cm) in postnatal week 4. The weekly increase in occipitofrontal circumference until a corrected age of 35 weeks’ gestation was 0.73 ± 0.20 cm (mean ± SD; median 0.74 cm; range 0.0–1.20 cm). At discharge, we observed a head circumference of 33.52 ± 1.89 cm (mean ± SD; median 33.50 cm; range 30.00–41.00 cm).

To ensure adequate growth over time and detect percentile crossing, an infant’s occipitofrontal circumference is routinely tracked on reference growth charts. For our study population, a head circumference percentile with median values of 40 (range 1–99) at birth and of 20 (range 1–90) at discharge was detected, demonstrating that many preterm infants are born with a small head circumference and do not show catch-up growth until hospital dismissal.

### Nutrition

Table 1 displays the nutritional composition of the neonatal diet. Analysis included the cumulative protein, fat, carbohydrate, and energy intake until postnatal days 14 and 28, respectively; the mean daily protein, fat, carbohydrate, and energy intake until a corrected age of 35 weeks of gestation; and the mean daily protein, fat, carbohydrate, and energy intake in each of the first four weeks of life.

**Table 1:** Protein, fat, carbohydrate, and energy intake during the indicated time periods

Variables	Mean ± SD	Median	Minimum	Maximum
Mean daily intake in the first week of life				
Carbohydrate, g/kg	11.20 ± 1.36	11.50	7.06	13.43
Fat, g/kg	3.31 ± 0.59	3.48	2.00	4.72
Protein, g/kg	2.92 ± 0.38	2.90	2.10	3.90
Energy, kcal/kg	88.21 ± 9.53	88.35	66.70	105.11
Mean daily intake in the second week of life				
Carbohydrate, g/kg	12.71 ± 1.79	12.89	7.46	16.62
Fat, g/kg	5.33 ± 0.98	5.55	1.63	6.95
Protein, g/kg	3.19 ± 0.72	3.06	1.91	4.66
Energy, kcal/kg	114.65 ± 14.85	116.36	71.74	146.82
Mean daily intake in the third week of life				
Carbohydrate, g/kg	13.62 ± 2.08	13.48	9.53	18.06
Fat, g/kg	5.87 ± 0.81	6.00	2.52	7.37
Protein, g/kg	3.34 ± 0.78	3.32	1.77	4.80
Energy, kcal/kg	123.89 ± 15.55	126.05	74.15	157.77
Mean daily intake in the fourth week of life (65 infants^a^)				
Carbohydrate, g/kg	14.36 ± 2.15	14.17	10.69	19.05
Fat, g/kg	6.17 ± 0.68	6.19	3.47	8.06
Protein, g/kg	3.43 ± 0.69	3.49	1.91	4.64
Energy, kcal/kg	130.22 ± 13.77	129.13	93.85	161.82
Cumulative intake until postnatal day 14				
Carbohydrate, g/kg	167.35 ± 18.39	170.31	101.65	195.23
Fat, g/kg	60.47 ± 9.41	62.04	35.76	77.44
Protein, g/kg	42.82 ± 6.98	41.67	28.77	58.61
Energy, kcal/kg	1420.02 ± 146.31	1447.03	969.09	1686.59
Cumulative intake until postnatal day 28 (65 infants^a^)				
Carbohydrate, g/kg	362.92 ± 35.58	361.93	288.67	445.63
Fat, g/kg	144.35 ± 16.06	146.73	91.90	181.61
Protein, g/kg	90.10 ± 15.70	89.63	56.10	119.89
Energy, kcal/kg	3193.52 ± 283.88	3219.31	2462.58	3904.21
Mean daily intake until a corrected age of 35 weeks’ gestation (65 infants^a^)				
Carbohydrate, g/kg	13.36 ± 1.40	13.03	10.14	17.45
Fat, g/kg	5.42 ± 0.61	5.52	3.22	7.97
Protein, g/kg	3.33 ± 0.59	3.28	2.02	4.82
Energy, kcal/kg	118.55 ± 11.10	119.88	88.24	154.25

^a^Two of the 67 patients were discharged before postnatal day 28 and a corrected age of 35 weeks’ gestation. *SD* standard deviation

### Deep gray matter volumes

The DGM volume at TEA was 18,865.55 ± 2980.31 mm^3^ (mean ± SD; median: 18,980.50 mm^3^; range: 10,901.70–35,029.26 mm^3^), and approximately two-thirds of the infants had a value between 17.000 and 21.000 mm^3^. These figures are comparable to the data of Srinivasan et al. [7–9]. Moreover, a significant association between DGM volume and birth weight was observed (*r* = 0.43886, *P* < 0.001).

### Lateral ventricular volumes

LV volumes showed a size difference, with left lateral ventricles often being considerably larger than right lateral ventricles. The left LV volume was 3508.18 ± 1958.03 mm^3^ (mean ± SD; median 2838.88 mm^3^; range 1501.30–10,606.40 mm^3^), whereas the right lateral ventricles had a volume of 2957.15 ± 1252.15 mm^3^ (mean ± SD; median 2772.90 mm^3^; range 1025.86–6254.40 mm^3^). The volumes of both lateral ventricles taken together ranged between 2697.64 and 16,860.80 mm^3^ (mean ± SD 6465.32 ± 2898.08 mm^3^; median 5338.10 mm^3^).

### Parameters with an impact on deep gray matter volume at term-equivalent age

Table 2 depicts the correlations between various nutritional components of the neonatal diet and DGM volume at TEA. All variables, which had been proven to be significant in these analyses, were considered for multiple linear regression analysis with the following results. One clinical and two nutritional variables were independently and significantly associated with DGM volume: head circumference at a corrected age of 35 weeks’ gestation (*P* < 0.001), mean daily fat intake in the fourth week of life (*P* = 0.004), and mean daily protein intake in the third week of life (*P* = 0.007). These parameters, however, differentially influence DGM volume. Head circumference at a corrected age of 35 weeks gestation and mean daily fat intake in the fourth week of life exhibited a positive correlation, whereas for mean daily protein intake in the third week of life, a negative correlation was observed. These relationships are expressed in the following equation:

**Table 2:** Correlation between nutrition and deep grey matter and lateral ventricular volume

Variables	Correlation between nutrition and deep grey matter volume		Correlation between nutrition and lateral ventricular volume	
	Coefficient of correlation	*P* value	Coefficient of correlation	*P* value
Mean daily intake in the first week of life				
Carbohydrate, g/kg	0.14433	0.244	− 0.31418	0.010
Fat, g/kg	0.21312	0.083	0.00546	0.965
Protein, g/kg	− 0.20349	0.099	0.05845	0.639
Energy, kcal/kg	0.20443	0.097	− 0.14381	0.246
Mean daily intake in the second week of life				
Carbohydrate, g/kg	0.09859	0.427	− 0.08181	0.510
Fat, g/kg	0.32201	0.008	0.00319	0.980
Protein, g/kg	− 0.30686	0.012	0.20491	0.096
Energy, kcal/kg	0.19014	0.123	0.00848	0.946
Mean daily intake in the third week of life				
Carbohydrate, g/kg	− 0.06370	0.609	0.05683	0.648
Fat, g/kg	0.31040	0.011	− 0.02367	0.849
Protein, g/kg	− 0.30878	0.011	0.13466	0.277
Energy, kcal/kg	0.06009	0.629	0.05553	0.655
Mean daily intake in the fourth week of life (65 infants^a^)				
Carbohydrate, g/kg	− 0.15875	0.207	0.10467	0.407
Fat, g/kg	0.40918	< 0.001	0.08965	0.478
Protein, g/kg	− 0.32211	0.009	0.07200	0.569
Energy, kcal/kg	0.02483	0.844	0.12738	0.312
Cumulative intake until postnatal day 14				
Carbohydrate, g/kg	0.14193	0.252	− 0.21828	0.076
Fat, g/kg	0.32764	0.007	0.00467	0.970
Protein, g/kg	− 0.29994	0.014	0.17070	0.167
Energy, kcal/kg	0.22827	0.063	− 0.05957	0.632
Cumulative intake until postnatal day 28 (65 infants^a^)				
Carbohydrate, g/kg	− 0.01090	0.931	− 0.05391	0.670
Fat, g/kg	0.42112	< 0.001	0.02456	0.846
Protein, g/kg	− 0.32905	0.007	0.13482	0.284
Energy, kcal/kg	0.15593	0.215	0.02920	0.817
Mean daily intake until a corrected age of 35 weeks’ gestation (65 infants^a^)				
Carbohydrate, g/kg	− 0.16854	0.180	0.17626	0.160
Fat, g/kg	0.30760	0.013	0.20496	0.102
Protein, g/kg	− 0.36727	0.003	0.19434	0.121
Energy, kcal/kg	0.00338	0.979	0.25037	0.044

^a^Two of the 67 patients were discharged before postnatal day 28 and a corrected age of 35 weeks’ gestation

$$\mathrm{DGM}=-11893-1019.87\bullet P3+1389.40\bullet F4+834.36\bullet \mathrm{HC}35$$

[with $\mathrm{DGM}$ = DGM volume (mL), $P3$ = mean daily protein intake in the third week of life (g/kg), $F4$ = mean daily fat intake in the fourth week of life (g/kg) and $\mathrm{HC}35$ = head circumference at a corrected age of 35 weeks of gestation [cm]].

Multiple regression analysis revealed a coefficient of determination *R*^*2*^ of 0.4978, indicating that nearly 50% of the variance in DGM volume can be explained by the combination of the three described variables.

Taken together, head circumference as well as protein and fat intake are associated with DGM volume and thus brain development following preterm birth.

Multiple regression analysis showed no significant association between DGM volume and the different clinical parameters of mode of feeding (parenteral and enteral, trophic feeding), hyperbilirubinemia requiring phototherapy, cerebral and intraventricular hemorrhages, small for gestational age (< 10th percentile), early onset infection (< 72 hours of life), late onset infection (> 72 hours of life), persistent ductus arteriosus (PDA), necrotizing enterocolitis (NEC), operation due to PDA, NEC or hernia and bronchopulmonary dysplasia.

### Parameters with impact on lateral ventricular volume at term-equivalent age

Analogous to DGM volume, potential correlations between different nutritional components and LV volume were examined (Table 2). For the lateral ventricular volume, all variables with a *P* value less than 0.10 in these analyses were considered for multiple linear regression analysis using forward selection. Two parameters, namely, the presence of intraventricular hemorrhage (*P* = 0.003) and mean daily carbohydrate intake in the first week (*P* = 0.038), were independently and significantly associated with LV volumes. The determination coefficient of this multiple model is *R*^2^ = 0.1919. Thus, the combination of both abovementioned parameters was only able to explain about 19% of the variance in LV volume.

The clinical parameters of mode of feeding (parenteral and enteral, trophic feading), hyperbilirubinemia requiring treatment, small for gestational age (< 10th percentile), early onset infection (< 72 hours of life), late onset infection (> 72 hours of life), persistent ductus arteriosus (PDA), necrotizing enterocolitis (NEC), operation due to PDA, NEC or hernia and bronchopulmonary dysplasia had no significant association with lateral ventricular volume at term-equivalent age.

## Discussion

Neurodevelopmental outcomes after preterm birth are influenced by a plethora of genetic and environmental factors. As a result, we used multiple linear regression analysis to simultaneously assess the impact of various clinical and nutritional parameters on the outcome variables in the present study. MRI-measured DGM volume at TEA was thereby positively correlated with head circumference at a corrected age of 35 weeks of gestation and mean daily fat intake in the fourth postnatal week, whereas mean daily protein intake in the third postnatal week showed a negative correlation. Taken together, the three abovementioned parameters were able to explain about 50% of the variance in DGM volume. Thus, in addition to occipitofrontal circumference and nutritional factors, other independent parameters are important to accurately predict neurodevelopmental outcome.

### Head circumference and deep gray matter volume

Head circumference has long been known as a marker for brain growth in preterm infants, and several studies have described a positive association with neurodevelopment [4, 10–12]. However, occipitofrontal circumference alone does not seem to sufficiently predict neurological outcome, and additional parameters, such as brain volumetrics obtained from cerebral MRI scans, have to be included for accurate prognosis [1]. The latter notion is supported by our own data showing that amplitude-integrated electroencephalography and/or cranial MRI are useful clinical diagnostic tools to assess the neurodevelopmental course of preterm infants [13].

The present study confirms the importance of occipitofrontal circumference in the prediction of neurological outcome, and its use in daily routine care still undisputedly represents a simple, rapid, and inexpensive clinical marker to evaluate brain size. Head circumference growth in turn is closely related to adequate nutrition of preterm infants [12, 14].

### Nutrition and deep gray matter volume

Key publications in recent years have emphasized that high energy and protein intake positively affects the neurological outcome of preterm infants. Conversely, insufficient nutrition after preterm birth is associated with slow growth (head circumference as well as weight), altered brain structure and impaired neurodevelopment [1, 3, 15]. In particular, very immature infants benefit from adequate nutrition; hence, Morgan et al. [4] advised offering neonates with a gestational age of < 29 weeks parenteral feeding with high energy and protein content. This recommendation was supported by a report from Keunen et al. [1] showing that an optimized macronutrient supply leads to sufficient head growth, optimal weight gain and increased axonal diameters in the corticospinal tract.

The caudate nucleus, a core part of the basal ganglia, is a cerebral region particularly sensitive to environmental influences. On one hand, postnatal malnutrition has been shown to have a negative impact on its development, and an association between reduced caudate volumes and learning and attention deficit disorders of preterm infants at school age was illustrated [2, 16]. On the other hand, van Beek et al. [17] observed significantly larger nuclei at TEA after optimized feeding, and Isaacs et al. [2] demonstrated a strong positive relationship between postnatal energy and protein intake, bilateral caudate size, and intelligence quotient in children born prematurely. Growth of the basal ganglia between birth and term was additionally positively correlated with faculty of speech and visual motor integration, highlighting this group of subcortical nuclei as a promising biomarker for neurodevelopmental outcome [18].

In the present study, we examined the association between DGM volume and nutrition in preterm infants and found a positive correlation with mean daily fat intake in the fourth postnatal week. Since the human brain has a high lipid content and fatty acids are essential for cell membrane and myelin synthesis, it is obvious that sufficient fat supply is necessary to enable adequate development of cerebral structures. Accordingly, recent studies reported that increased fat and energy provision in the first weeks of life leads to improved brain growth, accelerated white matter maturation, and increased DGM and cerebellar volume [8, 19]. Moreover, higher fat intake in preterm infants with a gestational age of < 30 weeks has been shown to be a protective factor with regard to brain lesions and dysmaturation and to improve cognitive and motor outcomes [5, 20]. However, fat supply also has to be balanced, as excessive supplementation results in altered adipose tissue distribution at TEA and adiposity, reduced brain volume and DGM size, and poor gyration [3, 7, 21–23]. Thus, further research must be conducted to define the optimal range of fat intake for preterm infants with different gestational ages and weights at birth.

Apart from the importance of adequate fat supply, increased protein supplementation in the first 28 days of life has also been associated with improved growth between birth and TEA [24]. Enteral protein intake seems to have beneficial effects on global brain, cerebellar, thalamic, and basal ganglia volume, which is reflected by an improved cognitive and motor outcome at two years of age [8, 25]. In contrast, we observed a negative relationship between mean daily protein intake in the third postnatal week and DGM volume at TEA. As the nutrition of preterm infants at our hospital is based on current guidelines by the ESPGHAN, the high protein content specified had already been considered and implemented in daily clinical routine before the onset of the present study. Consequently, only neonates with pronounced intrauterine malnutrition or gastrointestinal problems (e.g., multiple surgical procedures due to necrotizing enterocolitis) received even higher protein supplies as recommended. However, these patients are simultaneously considered a risk group for impaired brain development and poor neurological outcome, thus explaining the observed negative correlation between protein intake and DGM volume. This hypothesis is further supported by the fact that these infants require a longer period of parenteral nutrition due to critical illness and frequent intolerance of enteral feeding, which also negatively affects brain volume and white matter maturation [8]. Despite the adverse effect of protein supply on DGM growth observed in the present work, multiple studies—regardless of study design, time period examined, absolute protein content of nutrition, and route of administration—have independently proven that high protein intake is essential for adequate head circumference growth, normal brain development and favorable neurological outcome [25–28]. However, an effect on survival has not been described thus far.

Regarding carbohydrate intake, we did not observe any association with DGM volume. This finding is supported by a study from dit Trolli et al. [20], who could not find a correlation between carbohydrate intake following premature birth and neurological outcome at a corrected age of one year.

Sufficient energy supply is particularly essential for adequate brain development, as the human brain consumes nearly half of the energy offered in the first weeks of life [29]. Therefore, it is not surprising that postnatal energy intake is positively correlated with brain development, global brain, cerebellar, and DGM volume, and neurological outcome in the first year of life [20]. Preterm infants with energy deficits after birth often display reduced brain size and impaired neurodevelopment [1, 8, 19, 25]. In our study, we did not detect a relationship between energy intake and DGM volume at TEA. Possible explanations for these contradictory results include the inclusion of preterm infants with various gestational ages at birth, the examination of different time periods during the postnatal course, and the differences in nutritional guidelines between hospitals [25].

### Nutrition and lateral ventricular volume

In addition to the impact of nutrition on DGM volume, we also studied its relationship with LV size. Kesler et al. observed enlarged LV volumes in preterm infants and reported that lateral ventricular cerebrospinal fluid volume was significantly negatively correlated with subcortical gray volume in the preterm group. Additionally, the preterm group showed a significant negative correlation between occipital horn volume and occipital white matter volume and subcortical gray matter volume, which was not seen in term infants. The authors assumed that ventriculomegaly may in part be the result of subcortical tissue loss. With a special focus on volumes of occipital horns in preterm infants, the authors assume that local reductions in occipital lobe tissue further contributed to enlargement of this region aggravating the present loss of subcortical gray matter. In conclusion, these results described enlarged lateral ventricles as a consequence of developmental and pathologic processes [30]. Parikh et al. reported that the marked reductions in cerebral tissue and cerebral structures in high-risk extremely low birth weight preterm infants were accompanied by a compensatory increase in cerebrospinal fluid, indicating brain atrophy. Furthermore, they mentioned that loss of cortical and deep nuclear gray matter volume was less prominent than loss of cerebral white matter [22].

We found mean daily carbohydrate intake in the first postnatal week and intraventricular hemorrhage are independent predictors of LV volume. However, both parameters only predict approximately 19% of the variance in LV volume, illustrating that nutrition plays a role and other, yet unknown, factors contribute to the size of lateral ventricles.

Mean daily carbohydrate intake in the first postnatal week was negatively correlated with LV volume, which in turn implies that infants with low carbohydrate supply seem to have enlarged lateral ventricles. This finding can be explained by the clinical observation that very premature neonates usually show a reduced capability to metabolize carbohydrates and concomitantly have greater LV volumes and a higher risk of impaired neurological development than their term-born counterparts [25, 31]. The influence of intraventricular hemorrhage on LV volumes is known and depends on the grade of intraventricular hemorrhage. To date, there are no studies explicitly examining the relationship between nutrition and the size of lateral ventricles, but a positive effect on brain structure has been described [1–4, 14, 15, 32].

### Enteral feeding and brain structure

In the present study, mode of feeding (enteral and parenteral macronutrient intake, trophic feeding) showed no association with the examined brain structures. However, recent work showed a beneficial effect of enteral feeding on brain growth, as parenteral nutrition over a long period of time was associated with lower global brain, cerebellar, and deep and cortical gray matter volume [8, 19, 25, 33]. Additionally, early enteral feeding obviated the need for central venous access and was thus important to prevent catheter-associated infections [25]. The advantage of enteral nutrition over parenteral nutrition has been attributed to the close connection and reciprocal interactions between brain development, gut homoeostasis, and intestinal microflora. One potential pathophysiological mechanism is the existence of a microbiome-gut-brain-axis that supports the immature immune system by establishing an immunological balance and consequently reduces inflammation and protects against white matter injury [1]. Other studies reported a positive impact of circulating growth factors (e.g., insulin-like growth factor 1) on brain development and neurological outcome [1, 15].

### Limitations

The present study has several limitations. First, protein, fat, and carbohydrate concentrations in breast milk vary between individuals and are subject to changes during the postpartum course. Since the measurement of nutritional components in breast milk necessitates large sample volumes of 10 mL and these are, especially in the first days of life, urgently needed for the preterm infant, mean values were drawn from the current literature. Furthermore, this study was designed retrospectively with a limited number of patients, and thus, the results should be confirmed by a prospective multicentric study. This may additionally help to reduce the interindividual differences in breast milk due to a larger study population. As this study can provide evidence for important nutritional factors with an impact on neurologic structures, the relevance of early nutrition to brain development must be demonstrated by randomized controlled studies.

In conclusion, the present study highlights the importance of nutrition for brain development following premature birth. An early neonatal diet should therefore ideally consist of an optimal composition of proteins, carbohydrates, and fats, whereby adequate fat intake seems to have a beneficial effect on DGM volumes. To identify preterm infants with a high risk of neurodevelopmental impairment, head circumference can be routinely assessed as a simple marker to ensure adequate postnatal growth.

## Data Availability

The datasets generated and analyzed during the current study are available from the corresponding author on reasonable request.
